# Supplementary material for: Social Isolation Among Individuals with Incontinence: A Scoping Review
Source: Nurs Rep. 2025 Oct 24;15(11):375. doi: 10.3390/nursrep15110375 (PMC12655203; doi:10.3390/nursrep15110375)
Supplement: Supplementary file 1 [file nursrep-15-00375-s001.zip › nursrep-3912388-supplementary.pdf]

Supplementary Materials

Exploring Social Isolation Among Individuals with Incontinence: A Scoping Review

Table S1: Keywords and Search strategies

27.07.2024	PUBMED	(((("Social Isolation"[Mesh]) OR "Loneliness"[Mesh]) OR "Social Participation"[Mesh]) OR "Social Interaction"[Mesh]) AND (((("Ostomy"[Mesh]) OR "Surgical Stomas"[Mesh]) OR "Urinary Incontinence"[Mesh]) OR "Fecal Incontinence"[Mesh]),,,"(""Social Isolation""[MeSH Terms] OR ""Loneliness""[MeSH Terms] OR ""Social Participation""[MeSH Terms] OR ""Social Interaction""[MeSH Terms]) AND (""Ostomy""[MeSH Terms] OR ""Surgical Stomas""[MeSH Terms] OR ""Urinary Incontinence""[MeSH Terms] OR ""Fecal Incontinence""[MeSH Terms])"<br>**108 results**
03/08/24	CINAHL +PSYCINFO	TI (social isolation or loneliness or social exclusion) AND TI (incontinence or urinary incontinence or fecal incontinence)<br>**3 results**
03/08/24	CINAHL +PSYCINFO	TI (social isolation or loneliness or social exclusion) AND TI (ostomy or colostomy or ileostomy or stoma)<br>**1 results**

Table S2. Relevance of Social Isolation in the Included Records [26–37,39–43,45–49]

Title	Main research focus on social isolation	Supplementary focus on social isolation
Experiences of women and men living with urinary incontinence: A phenomenological study (Esparza et al., 2018)		X
The trajectory of hope and loneliness in rectal cancer survivors with major low anterior resection syndrome: A qualitative study (Pape et al., 2022)	X	
“I am a person but I am not a person”: experiences of women living with obstetric fistula in the central region of Malawi (Changole et al., 2017)		X
Psychological resilience and active social participation among older adults with incontinence: a qualitative study (Takahashi et al., 2016)	X	
Female Urinary Incontinence in China: Experiences and Perspectives (Komorowski & Chen, 2006)		X
The Management of Urinary Incontinence by Community-Living Elderly (Mitteness, 1987)		X
A qualitative exploration of the experiences of children with spina bifida and their parents around incontinence and social participation (Fischer et al., 2015)	X	
Problematic aspects of faecal incontinence according to the experience of adults with spina bifida (Johnsen et al., 2009)	X	

Urinary Incontinence, Mental Health, and Loneliness Among Community-Dwelling Older Adults in Ireland (Stickley et al., 2017)	X	
Effects of stigma on Chinese women's attitudes towards seeking treatment for urinary incontinence (Wang et al., 2015)		X
Urinary Incontinence and Depressive Symptoms: The Mediating Role of Physical Activity and Social Engagement (Park et al., 2022)	X	
Linking stigma to social isolation among colorectal cancer survivors with permanent stomas: the chain mediating roles of stoma acceptance and valuable actions (Li et al., 2024)	X	
Sense of Coherence as a Key to Improve Homebound Status Among Older Adults with Urinary Incontinence (Takahashi et al., 2015)	X	
Stigma in patients with rectal cancer: a community study (MacDonald & Anderson, 1984)		X
Self-Reported Social and Emotional Impact of Urinary Incontinence (Fultz & Herzog, 2001)	X	
Urinary incontinence and loneliness in Canadian seniors (Ramage-Morin & Gilmour, 2013)	X	
The association between urinary and fecal incontinence and social isolation in older women (Yip et al., 2013)	X	
Social Connectivity in Those 24 Months or Less Postsurgery (Nichols, 2011)	X	
Disease stigma and its mediating effect on the relationship between symptom severity and quality of life among community-dwelling women with stress urinary incontinence: a study from a Chinese city (Wan et al., 2014)		X
Incontinence and loneliness among Chinese older adults with multimorbidity in primary care: A cross-sectional study (Wang et al., 2015)	X	
Living With an Intestinal Stoma: A Qualitative Systematic Review (Capilla-Díaz et al., 2019)		X
Perceptions and help-seeking behaviours among community-dwelling older people with urinary incontinence: A systematic integrative review (Yan et al., 2022)		X
Understanding the health and social care needs of people living with IBD: A meta-synthesis of the evidence (Kemp, 2012)	X	
